# Efficient Hydroxyl Diffusion Triggers Surface Hydronium Enrichment and Bulk Proton Participation to Boost Alkaline Hydrogen‐Evolving Reaction

**DOI:** 10.1002/adma.74662

**Published:** 2026-08-17

**Authors:** Daqin Guan, Hengyue Xu, Xiao Sun, Leqi Zhao, Chun‐Kuo Peng, Chao Jing, Yu‐Cheng Huang, Jiayi Tang, Nai Shi, Zezhou Lin, Xiaomin Xu, Zhiwei Hu, Zongping Shao

**Affiliations:** ^1^ Curtin Centre For Advanced Energy Materials and Technologies (CAEMT) Western Australian School of Mines (WASM) Curtin University Perth Western Australia Australia; ^2^ Department of Chemistry Tsinghua University Beijing China; ^3^ Geoscience Atom Probe Facility John de Laeter Centre Curtin University Perth Western Australia Australia; ^4^ Key Laboratory of Interfacial Physics and Technology Shanghai Institute of Applied Physics Chinese Academy of Sciences Shanghai China; ^5^ Department of Applied Physics The Hong Kong Polytechnic University Hong Kong China; ^6^ Max‐Planck‐Institute for Chemical Physics of Solids Dresden Germany

**Keywords:** alkaline hydrogen‐evolving reaction, anion‐exchange‐membrane electrolyzer, bulk hydroxyl diffusion, bulk proton participation, surface hydronium enrichment

## Abstract

Deciphering the diffusion, induction, and reaction processes of key chemical species in the catalyst lattice is critical for solution‐phase electrochemical applications, yet remains underexplored. Taking the alkaline hydrogen‐evolving reaction (HER) in an anion‐exchange‐membrane (AEM) electrolyzer as an example, prior efforts were devoted to optimizing water dissociation and proton recombination steps on catalyst surfaces, neglecting the important role of bulk electrochemistry induced by abundant OH^−^ in the electrolyte. As a proof‐of‐concept, we design oxygen‐vacancy‐ordered and oxygen‐vacancy‐disordered model oxides to explore the bulk electrochemistry triggered by OH^−^ diffusion. Combined systematic experiments and computations reveal that the ordered and high‐concentration features of oxygen vacancies improve the mobility and flux of OH^−^ diffusion into the bulk lattice, respectively. Multiple operando characterizations demonstrate that efficient bulk OH^−^ diffusion lowers surface OH^−^ concentration and thus drives the water ionization equilibrium toward products (2H_2_O ↔ H_3_O^+^ + OH^−^) following Le Chatelier's principle, contributing to enriched surface H_3_O^+^ and enhanced surface HER kinetics. Interestingly, due to the electrostatic interactions, bulk OH^−^ diffusion behavior triggers surface‐to‐bulk proton migration and participation, extending HER regions from the surface to the bulk and thus boosting HER activity. The high OH^−^ diffusion capability of the cathode also greatly improves the AEM‐electrolyzer performance. Our work offers new insights into the long‐overlooked bulk electrochemistry.

## Graphical abstract

For the first time, we shed light on the surface‐to‐bulk diffusion, induction, and reaction behaviors of OH^−^, H^+^, and H_2_O species on model oxides for alkaline hydrogen‐evolving reaction (HER). We first demonstrate that bulk OH^−^ diffusion significantly affects the generation of proton sources and the diffusion/reaction behaviors of protons, thus governing the performance of alkaline HER and anion‐exchange‐membrane (AEM) electrolyzers.

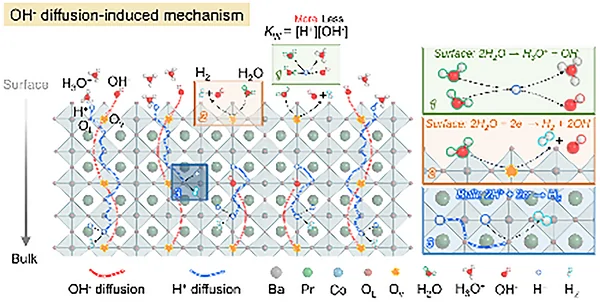

## 1 Introduction

The diffusion behaviors of key chemical species in the bulk of catalysts play important roles in multiple electrochemical applications. For example, the (de)intercalation of Li^+^/Na^+^/K^+^/NH_4_^+^ ions within the bulk of anodes and cathodes in Li^+^/Na^+^/K^+^/NH_4_^+^‐ion batteries greatly affects the charge–discharge performance [1, 2]. Additionally, in high‐temperature solid oxide cells (e.g., protonic ceramic fuel cells (PCFC) and solid oxide fuel cells (SOFC)), the H^+^/O^2−^ diffusion capabilities of ceramic lattices govern the cells’ efficiency [3, 4]. Moreover, in electrochromic smart windows, the insertion and extraction behaviors of Li^+^/H^+^ ions within WO_3_, V_2_O_5_, and NiO lattices determine the windows’ optical modulation, response time, and energy efficiency [5, 6]. However, for electrocatalysis reactions in solutions, the literature generally focused on surface catalysis processes [7, 8], neglecting the critical influence of bulk electrochemistry, for example, bulk ion diffusion, induction, and reaction.

As a typical case, the alkaline hydrogen‐evolving reaction (HER, 2H_2_O + 2e^−^ → H_2_ + 2OH^−^) is a pivotal part of the anion‐exchange‐membrane (AEM) electrolyzer for sustainable green hydrogen production (Figure 1a) [9, 10]. Prior studies generally emphasized the surface electrochemistry at the catalyst‐electrolyte interfaces, that is, the key steps of surface water dissociation (Volmer step: H_2_O → H^*^ + OH^*^) and surface proton recombination for H_2_ generation (Heyrovsky step: H_2_O + H^*^ → H_2_ + OH^*^; Tafel step: 2H^*^ → H_2_) [11, 12, 13, 14]. Through designing specific active sites for both H_2_O and H^*^ species, surface synergetic effects can be realized to boost HER activity. For example, in the case of multiphase Ni(OH)_2_‐Pt composites, surface Ni(OH)_2_ and Pt phases serve as active sites for water dissociation and proton recombination, respectively [15]. Moreover, in the single‐phase Gd_0.5_La_0.5_BaCo_2_O_6‐x_ oxide, surface oxygen vacancies benefit water dissociation, while surface metallic Co^4+^ ions enable efficient proton recombination [16]. Nevertheless, as the abundant ions in alkaline electrolyte, the effects of surface‐to‐bulk OH^−^ diffusion and induction on the HER and AEM electrolyzer performance are always overlooked (Figure 1a). Furthermore, as catalysis proceeds, the reactive regions gradually extend from the surface to the bulk of the catalyst lattice (e.g., lattice oxygen/proton participation) [17, 18]. Therefore, shedding light on the diffusion, induction, and reaction behaviors of key chemical species (OH^−^, H^+^, and H_2_O here) in the catalyst lattice will greatly help establish structure‐activity relationships and understand performance origins, offering important guidance for the rational optimization in electrochemical reactions and devices. Nonetheless, this remains a challenging but critical blind spot.

**FIGURE 1:**
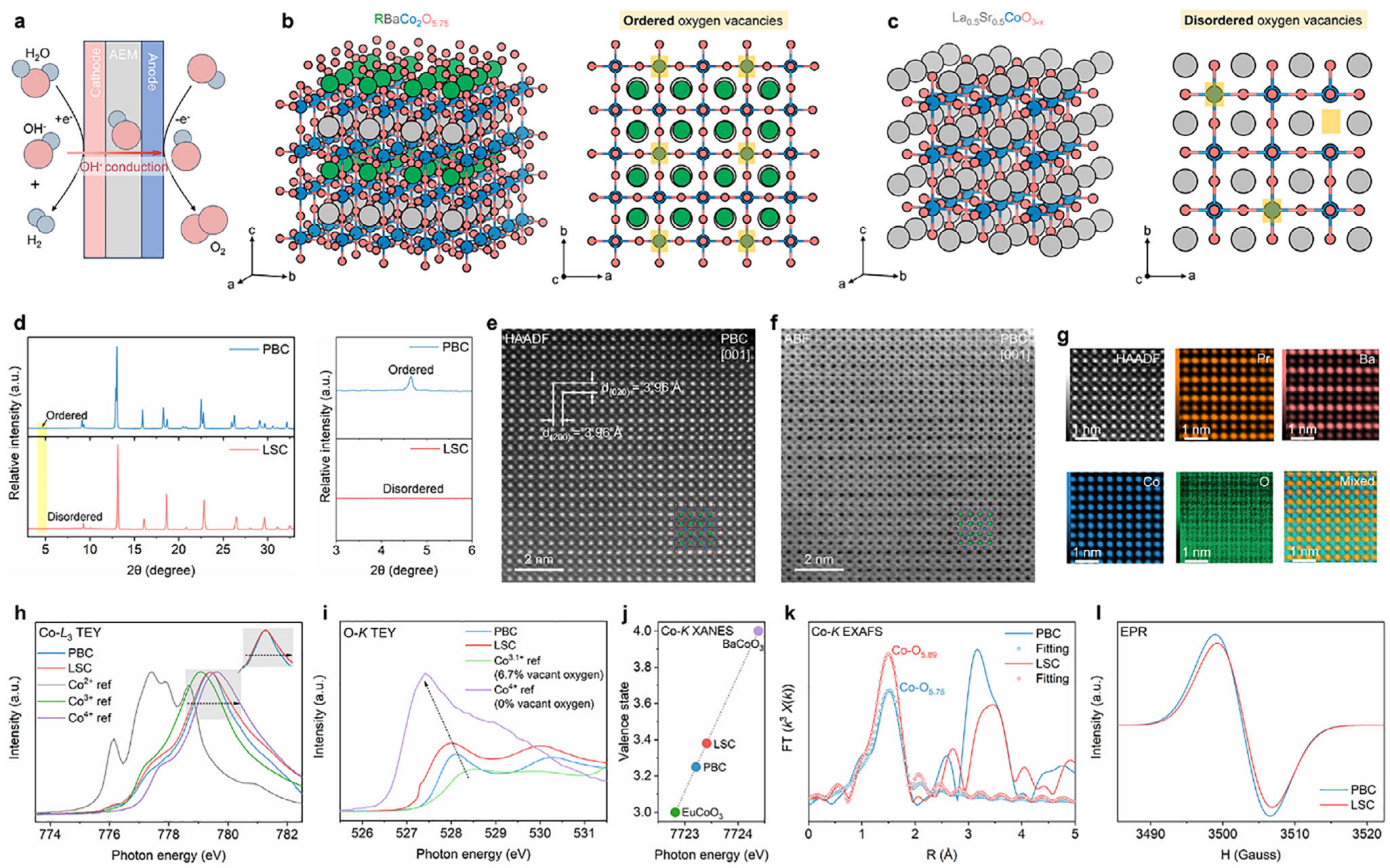
Characterizations of the ordering and content of oxygen vacancies. (a) The neglected role of OH^−^ conduction of cathode in AEM device. The molecular structures and the ordering of oxygen vacancies of (b) RBaCo_2_O_5.75_ and (c) La_0.5_Sr_0.5_CoO_3‐x_. (d) SXRPD patterns of PBC and LSC. The spherical‐aberration‐corrected (e) HAADF image, (f) ABF image, and (g) atomic mapping for PBC. (h) The Co‐*L*_3_ and (i) O‐*K* XAS plots in TEY mode for PBC and LSC, where CoO, Sr_2_CoO_3_Cl, and SrCoO_3_ single crystals were used as pure Co^2+^, pure Co^3+^, and pure Co^4+^ standards, respectively. Inset (h) is the enlarged view of the Co‐*L*_3_ absorption peak region. The results of (j) Co‐*K* XANES and (k) Co‐*K* EXAFS spectra. (l) EPR plots for PBC and LSC.

Here, we take the view that bulk OH^−^ diffusion could significantly affect the performance of alkaline HER and AEM devices. Considering that negative OH^−^ ions tend to adsorb on positive oxygen vacancies, we explore the influence of bulk OH^−^ diffusion in oxygen‐vacancy‐ordered and oxygen‐vacancy‐disordered model oxides. Combined OH^−^ conductivity measurement, sputtering X‐ray photoelectron spectroscopy (XPS), time of flight secondary ion mass spectrometry (ToF‐SIMS), and density functional theory (DFT) calculations uncover that the ordered oxygen vacancies facilitate OH^−^ diffusion mobility, while the high‐concentration oxygen vacancies improve OH^−^ diffusion flux. By integrating operando Fourier transform infrared (FTIR), operando Raman, isotope labeling experiments, and computations, we discover that surface OH^−^ concentration is reduced due to the efficient bulk OH^−^ diffusion, shifting the water ionization equilibrium toward products (2H_2_O ↔ H_3_O^+^ + OH^−^) under Le Chatelier's principle and thus creating a unique acid‐like surface environment with enriched H_3_O^+^ to accelerate surface HER kinetics. Noteworthily, under the charge induction effect, protons from surface H_3_O^+^ and surface water dissociation step migrate into the bulk lattice along with bulk OH^−^ diffusion to trigger an exceptional bulk proton participation pathway. Because of the steric hindrance in the catalyst structure, relatively large H_2_O molecules are confined to surface dissociation. Given that OH^−^ migrates from the cathode across the membrane and further to the anode reaction (Figure 1a), efficient bulk OH^−^ diffusion of cathode also significantly enhances the AEM electrolyzer performance. Our study opens a new avenue for the long‐overlooked bulk electrochemistry in solution‐phase electrochemical reactions and applications.

## 2 Results and Discussion

### 2.1 Characterizations of the Ordering and Content of Oxygen Vacancies

The ordering and content are the major properties of oxygen vacancies in catalysts. Among various material systems, perovskite oxides (ABO_3‐x_, *A* = alkaline/rare‐earth metal ions, *B* = transition metal ions, *x* = oxygen‐vacancy stoichiometry) possess abundant structural features to enable the facile design of oxygen vacancies [19, 20]. It has been widely acknowledged that A‐sites mainly act as the structural environment surrounding B–O active sites, which exhibit stable physicochemical properties and do not directly participate in electrochemical reactions [21, 22]. In B–O active sites, the properties of B‐sites are strongly correlated with those of O‐sites or oxygen vacancies. Since the A‐sites possess stable and fixed valence states, the overall charge neutrality of ABO_3‐x_ is actually maintained through the valence‐state balance between B‐sites and O‐sites/vacancies [21, 22]. An increase in oxygen‐vacancy concentration corresponds to a decrease in the number of oxygen atoms, namely, a reduction in the total negative charges in ABO_3‐x_. To maintain charge neutrality, the average valence state of B‐sites is correspondingly reduced, leading to a decrease in the total positive charges. Therefore, the ordering and concentration of oxygen vacancies in the perovskite structure can effectively reflect the properties of B–O active sites, which can thus be regarded as dominant factors governing the performance of perovskite oxides.

Extensive screening shows that the arrangement of oxygen vacancies in cation‐ordered *R*BaCo_2_O_5.75_ (*R* = lanthanides) double perovskite (along the lanthanide plane as indicated by the yellow squares in Figure 1b) is perfectly ordered [23, 24], while that in cation‐disordered ABO_3‐x_ single perovskite (yellow squares in Figure 1c) is disordered [25, 26]. The ordered oxygen vacancy in RBaCo_2_O_5.75_ is realized by the large difference in ionic radius between R^3+^ and Ba^2+^ and the optimal oxygen‐vacancy content [16, 23]. We thus synthesized PrBaCo_2_O_6‐x_ (PBC with 1.32 Å Pr^3+^ and 1.61 Å Ba^2+^ cations) double perovskite and La_0.5_Sr_0.5_CoO_3‐x_ (LSC with 1.36 Å La^3+^ and 1.44 Å Sr^2+^ ions) single perovskite as the model oxides via the conventional sol–gel method to investigate the roles of bulk OH^−^ diffusion. Laboratory x‐ray diffraction (XRD) spectra and related structural refinements show that both PBC and LSC are pure phases with space groups of *P*4*/mmm* and *Pm*‐3*m*, respectively (Figure S1 and Table S1).

First, we characterize the ordering of oxygen vacancies in the model oxides. It has been reported that the oxygen‐vacancy ordering can be reflected by the synchrotron x‐ray powder diffraction (SXRPD) spectrum, where an ordered configuration gives rise to an additional Bragg peak at low angle [23]. The SXRPD spectrum of PBC exhibits a distinct diffraction peak at ∼4.65 ° using a wavelength (*λ*) of 0.6199 Å (Figure 1d), according well with the reported ordered oxygen vacancies at ∼3.38 ° under *λ* = 0.4503 Å [23]. However, no Bragg peaks were found at low angles in the SXRPD spectrum of LSC (Figure 1d), indicating its disordered oxygen vacancies in the lattice. The spherical‐aberration‐corrected scanning transmission electron microscopy (STEM) was further applied to study the atomic distribution of PBC, where high‐angle annular dark‐field (HAADF) and annular bright field (ABF) modes are sensitive to metal and oxygen atoms, respectively. Under the HAADF imaging mode, two interplanar distances of 3.96 Å are indexed to the (020) and (200) planes of PBC (Figure 1e), matching well with XRD refinements. The corresponding view along the [001] zone‐axis direction of the PBC structure is presented in the inset of Figure 1e. Under the ABF imaging mode, the bright and dark regions are oxygen and metal atoms, respectively [27]. As illustrated in Figure 1f, the arrangement of oxygen atoms in PBC along [001] zone axis is ordered. The ordered distribution of all atoms in PBC can also be evidenced by its atomic mapping (Figure 1g). Moreover, the images of HAADF and atomic mapping along [10$\bar{1}$] zone axis further verify the ordered configuration of PBC (Figure S2). In contrast, the spherical‐aberration‐corrected ABF and HAADF images, together with atomic mapping, confirm the disordered distribution of oxygen atoms in LSC (Figure S3), further indicating its disordered arrangement of oxygen vacancies.

Then, we analyze the content of oxygen vacancies in the model oxides. Owing to charge neutrality in the material lattice, more oxygen vacancies indicate fewer lattice oxygen atoms, namely, fewer negative charges, leading to a lower valence state of the transition metal cations. Therefore, the oxygen‐vacancy content can be inferred from the Co valence state of model oxides. It has been acknowledged that soft x‐ray absorption spectroscopy (XAS) under the total electron yield (TEY) mode is highly sensitive to the surface Co valence, where a positive energy shift of the weight of Co‐*L*_3_ absorption peak suggests a higher Co valence as verified by the spectra of pure Co^2+^, pure Co^3+^, and pure Co^4+^ standards [16, 28, 29, 30] in Figure 1h. The weight of Co‐*L*_3_ absorption peaks of both PBC and LSC lies between pure Co^3+^ and pure Co^4+^ standards, suggesting their mixed Co^3+/4+^ valence. Besides, compared with PBC, the weight of Co‐*L*_3_ absorption peak of LSC shifts to higher energy, revealing a lower surface Co valence (namely, more surface oxygen vacancies) in PBC. Additionally, in Co 2*p* XPS spectra, the binding energy of Co 2*p* peak for PBC is lower than that for LSC (Figure S4), further confirming the lower surface Co valence and more surface oxygen vacancies in PBC. It has been recognized that a negative energy shift accompanied by increased spectral weight of the pre‐edge peak in O‐*K* XAS spectra under the TEY mode means enhanced surface Co─O covalence and increased surface Co valence [16, 30, 31]. Relative to LSC, the pre‐edge peak of O‐*K* XAS spectrum for PBC appears at higher energy with reduced spectral weight (Figure 1i), demonstrating the weakened surface Co─O covalency and lower surface Co valence (namely, more surface oxygen vacancies) in PBC. To enable a clearer comparison of oxygen‐vacancy concentrations based on the O‐*K* XAS spectra, we further introduced two reference standards, namely, GdBaCo_2_O_5.6_ (Co^3.1+^) with ∼6.7% vacant oxygen sites and SrCoO_3_ (pure Co^4+^) without oxygen vacancies [29, 32]. As shown in Figure 1i, the absorption feature exhibits a gradual negative energy shift from GdBaCo_2_O_5.6_ to PBC, LSC, and further to SrCoO_3_, together with a gradual increase in spectral weight. This trend confirms that the oxygen‐vacancy concentration decreases in the order of GdBaCo_2_O_5.6_ (∼6.7% vacant oxygen sites) > PBC > LSC > SrCoO_3_ (0% vacant oxygen sites). In O 1*s* XPS spectra, the signal at ∼528.5 eV for perovskite oxide is indexed to the lattice oxygen [33, 34]. The intensity of lattice‐oxygen XPS signal in PBC is lower than that in LSC (Figure S5), further strengthening our conclusions.

To investigate the oxygen‐vacancy content in the bulk of model oxides, we conducted measurements of Co‐*K* XAS of near‐edge structure (XANES), Co‐*K* XAS extended x‐ray absorption fine structure (EXAFS), and electron paramagnetic resonance (EPR). It has been widely acknowledged that a positive energy shift of the normalized absorption of ∼0.8 in the Co‐*K* XANES spectrum indicates an increased bulk Co valence as revealed by the spectra of pure Co^3+^ and pure Co^4+^ standards [35, 36, 37] in Figure S6. The normalized absorption of ∼0.8 in the Co‐*K* XANES spectra for both PBC and LSC falls between pure Co^3+^ and pure Co^4+^ standards, demonstrating their mixed Co^3+/4+^ valence. Further valence calculations via linear interpolation with the standards [35, 37] show that the bulk Co valence of PBC and LSC is 3.25+ and 3.38+ (Figure 1j), respectively, corroborating more bulk oxygen vacancies in PBC. According to the charge neutrality rule, we can now identify the specific molecular formula of PBC and LSC as PrBaCo_2_O_5.75_ and La_0.5_Sr_0.5_CoO_2.94_, respectively. The Co‐*K* EXAFS spectrum is powerful for resolving the bulk local structure. As shown in Figure 1k, the first peak at ∼1.5 Å is the Co─O shell, where a reduced intensity of Co─O shell means decreased Co─O coordination numbers, namely, increased oxygen vacancies [34, 38]. As expected, the intensity of Co─O shell for PBC is lower than that for LSC, indicating lower Co─O coordination numbers and more bulk oxygen vacancies in PBC. The Co‐*K* EXAFS fitting results in Table S2 further reveal a lower Co─O coordination number in PBC (5.75, corresponding to ∼4.2% vacant oxygen sites) than in LSC (5.89, corresponding to ∼1.8% vacant oxygen sites), further confirming the higher oxygen‐vacancy concentration in PBC. To directly measure the bulk oxygen vacancies, we conducted oxygen‐vacancy‐sensitive EPR, where an increase in the intensity at *g* = 2 means larger oxygen‐vacancy content [38]. Compared with LSC, the EPR intensity at *g* = 2 of PBC is higher (Figure 1l), confirming more bulk oxygen vacancies in PBC.

We can conclude from the above results that relative to oxygen‐vacancy‐disordered LSC, the oxygen‐vacancy‐ordered PBC exhibits more surface‐to‐bulk oxygen vacancies. The bulk OH^−^ diffusion behaviors in the two model oxides will then be explored.

### 2.2 Hydroxyl Conductivity and Diffusion

The key chemical species in alkaline HER include OH^−^, H^+^, and H_2_O. Considering that OH^−^ is abundant in alkaline electrolyte, we first investigate the OH^−^ diffusion capability of the model oxides. To measure the OH^−^ conductivity, we used OH^−^‐conducting AEM on both sides of the calcined dense oxide pellets to block H^+^ transport and electron conduction in alkaline solutions (Figure 2a). It should also be noted that high‐temperature calcination is necessary to obtain dense oxide pellets and eliminate physical pores, which could otherwise affect the accuracy of the OH^−^ conductivity measurements of the catalysts. The OH^−^ conductivity (*p*) of model oxides can be calculated as *p* = *L*/(*S*×*R*), where *L*, *S*, and *R* denote the pellet thickness, the OH^−^‐conducting area, and the measured ohmic resistance, respectively (Figure S7). Notably, the measured OH^−^ conductivity of PBC (4.84 × 10^−4^ S cm^−1^) is ∼3.64 times that of LSC (1.33 × 10^−4^ S cm^−1^) (Figure 2b), suggesting the superior OH^−^ diffusion capability/mobility of PBC. The ordered oxygen vacancies in PBC endow it with short OH^−^ diffusion pathways for accelerated OH^−^ diffusion mobility.

**FIGURE 2:**
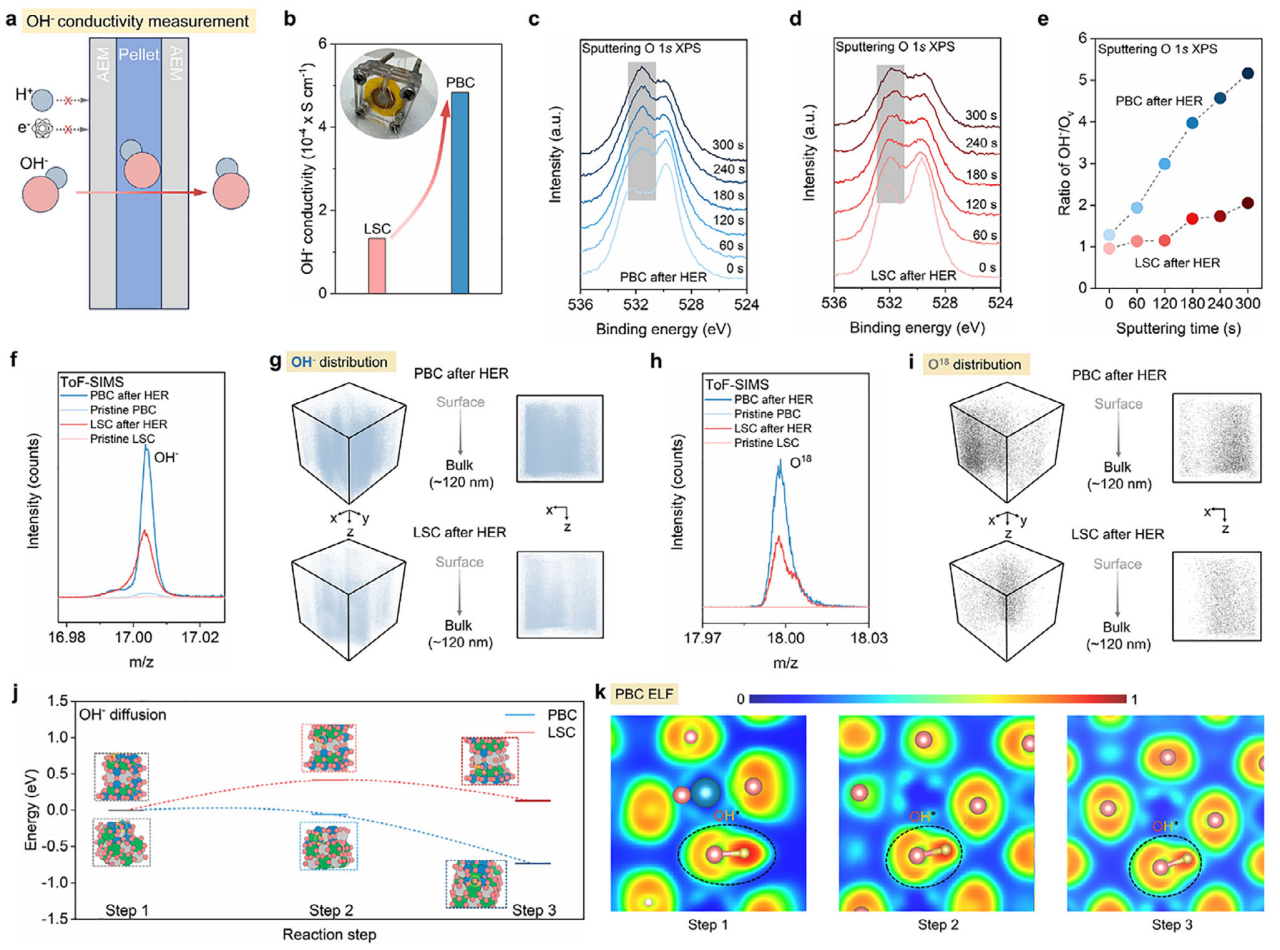
Hydroxyl conductivity and diffusion. (a) The measurement method of OH^−^ conductivity. (b) The OH^−^ conductivities of PBC and LSC, where inset is the setup. The sputtering O 1*s* XPS spectra of (c) PBC after HER and (d) LSC after HER. (e) The ratio of OH^−^/*O*_v_ calculated from the fitting results of sputtering O 1*s* XPS spectra for PBC after HER and LSC after HER. (f) The ToF‐SIMS peak signals of OH^−^ species for model oxides before and after HER, and (g) related 3D mappings for model oxides after HER. (h) The ToF‐SIMS peak signals of O^18^ species for model oxides before and after HER, and (i) related 3D mappings for model oxides after HER. (j) The calculated adsorption energies of OH^−^ from the surface to the bulk. (k) ELF plots of the adsorption of OH^−^ from the surface to the bulk for PBC.

Owing to the superior OH^−^ conductivity of PBC, a high surface‐to‐bulk flux of OH^−^ species should be observed in PBC as compared with LSC, where high‐concentration oxygen vacancies in PBC could provide more transport sites for OH^−^ diffusion. Herein, we performed sputtering XPS and ToF‐SIMS to examine the surface‐to‐bulk OH^−^ flux in model oxides after alkaline HER. Both sputtering XPS and ToF‐SIMS use an etching method to detect material properties at different depths, where the probing depth increases with increasing etching time [19, 30, 38]. First, we measured the O 1*s* XPS spectra at the etching time of 0, 60, 120, 180, 240, and 300 s (depth of ∼15 nm). As shown in Figure 2c,d, the characteristic signal of OH^−^ at ∼531.8 eV [33, 34] for both PBC and LSC after alkaline HER enhances with increasing etching time. Relative to LSC after HER, the enhancement of OH^−^ signal for PBC after HER is more pronounced, implying more OH^−^ species. To precisely extract the OH^−^ content, we fitted the sputtering O 1*s* XPS spectra with three components, namely, lattice oxygen at ∼528.5 eV, oxygen vacancy at ∼529.7 eV, and OH^−^ species at ∼531.8 eV, following previous literature [33, 34, 39]. The fitting results show that with increasing etching time, the content of OH^−^ species increases while oxygen‐vacancy (*O*_v_) content decreases for both PBC and LSC after HER (Figures S8 and S9), proving that OH^−^ diffuses through oxygen vacancies. By comparing the OH^−^/*O*_v_ ratio, we can evaluate the OH^−^ diffusion capability of the model oxides. At different probing depths, the OH^−^/*O*_v_ ratio of PBC after HER is much higher than that of LSC (Figure 2e), indicating the superior OH^−^ diffusion capability of PBC.

Then, to further compare the OH^−^ diffusion flux in deeper regions, we performed ToF‐SIMS on model oxides after alkaline HER, where the probing depth is ∼120 nm. The intensity of OH^−^ signal with *m/z* ≈ 17 for PCB after HER is significantly higher than that for LSC (Figure 2f), supported by the denser 3D OH^−^ distribution in PCB after HER from the surface to the bulk (Figure 2g). We also conducted ToF‐SIMS on model oxides after HER in H_2_O^18^‐containing alkaline solutions. Since H_2_O^18^ is dissociated into H^+^ and O^18^H^−^ (*m/z* ≈ 19, overlap with F^−^ in the binder) during HER, the tracking of O^18^ (*m/z* ≈ 18.00, distinguishable from H_2_O with *m/z* ≈ 18.01) spatial distribution on model oxides after HER will also help compare the hydroxyl diffusion flux. As expected, the intensity of O^18^ signal (Figure 2h) and the density of 3D surface‐to‐bulk O^18^ distribution (Figure 2i) for PBC after HER are higher than those for LSC. These findings reinforce the conclusion that PBC possesses stronger bulk OH^−^ diffusion capability than LSC.

Furthermore, we calculated the adsorption energies of OH^−^ diffusion (steps 1∼3) in model oxides to provide theoretical insights, where all the calculated energies of PBC are lower than those of LSC (Figure 2j). This indicates that the energy barriers of OH^−^ diffusion in PBC are lower than those in LSC, enabling easier surface‐to‐bulk OH^−^ diffusion in PBC. In addition, the electron localized function (ELF) calculations can further display the binding strength with adsorbates, where the regions with higher intensity show stronger absorption with adsorbates [40, 41]. ELF maps uncover that the OH^−^absorption from step 1 to step 3 for PBC gradually weakens (Figure 2k), while that for LSC remains consistently strong (Figure S10), demonstrating the facile bulk OH^−^ diffusion in PBC.

### 2.3 Surface Hydronium Enrichment and Bulk Proton Diffusion

Given the high abundance of OH^−^ in alkaline electrolyte, the bulk OH^−^ diffusion into the material lattice could affect the transport and reaction behaviors of other important species, for example, the key H^+^ in alkaline HER. To examine the ion adsorption/desorption dynamics at material surfaces, we conducted operando FTIR and operando Raman. With increasing HER overpotentials, the signals at ∼2900, ∼2980, and ∼3670 cm^−1^ of operando FTIR on PBC become stronger (Figures 3a and S11), which can be assigned to the appearance of H_3_O^+^ species as reported [42, 43]. The superior bulk OH^−^ diffusion capability of PBC could lower surface OH^−^ content and thus shift the water ionization equilibrium toward products (2H_2_O ↔ H_3_O^+^ + OH^−^) (point 1 in Figure 4d), contributing to surface H_3_O^+^ enrichment. This unique acid‐like surface local environment can provide more protons from enriched H_3_O^+^ species to facilitate surface HER kinetics as compared with the alkaline environment. Limited by the poor bulk OH^−^ diffusion capability, the OH^−^ species (characteristic FTIR signal at ∼3350 cm^−1^) [30, 38] are blocked at the surface of LSC (Figures 3b and S12), leading to sluggish surface HER kinetics. Moreover, operando Raman further verifies that as the HER overpotential increases, PBC shows a characteristic band of H_3_O^+^ at ∼1700 cm^−1^ (Figure 3c) [44, 45], which is absent for LSC (Figure 3d).

**FIGURE 3:**
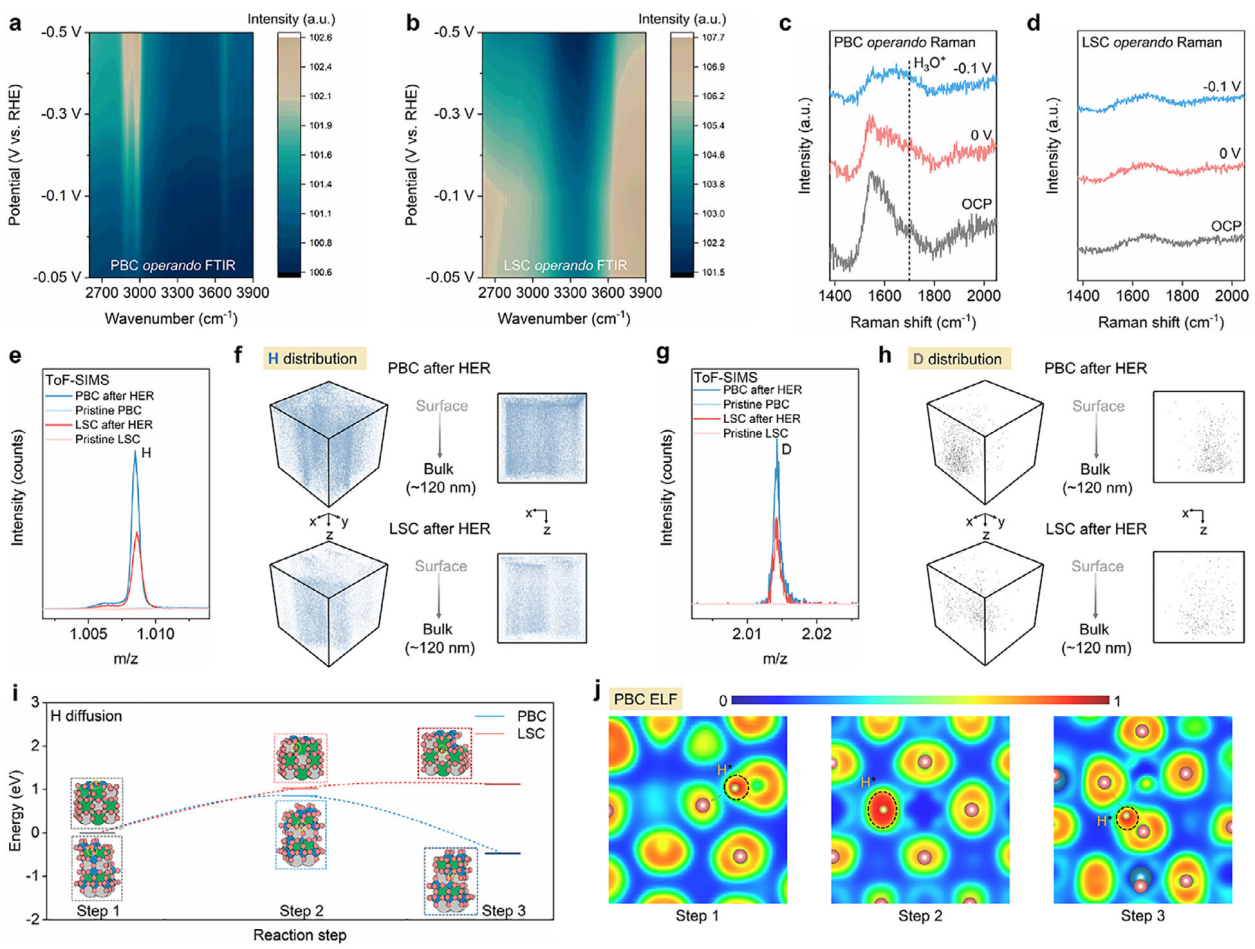
Surface hydronium enrichment and bulk proton diffusion. Operando FTIR of (a) PBC and (b) LSC. Operando Raman of (c) PBC and (d) LSC. (e) The ToF‐SIMS peak signals of H^+^ species for model oxides before and after HER, and (f) related 3D mappings for model oxides after HER. (g) The ToF‐SIMS peak signals of D species for model oxides before and after HER, and (h) related 3D mappings for model oxides after HER. (i) The calculated adsorption energies of H^+^ from the surface to the bulk. (j) ELF plots of the adsorption of H^+^ from the surface to the bulk for PBC.

**FIGURE 4:**
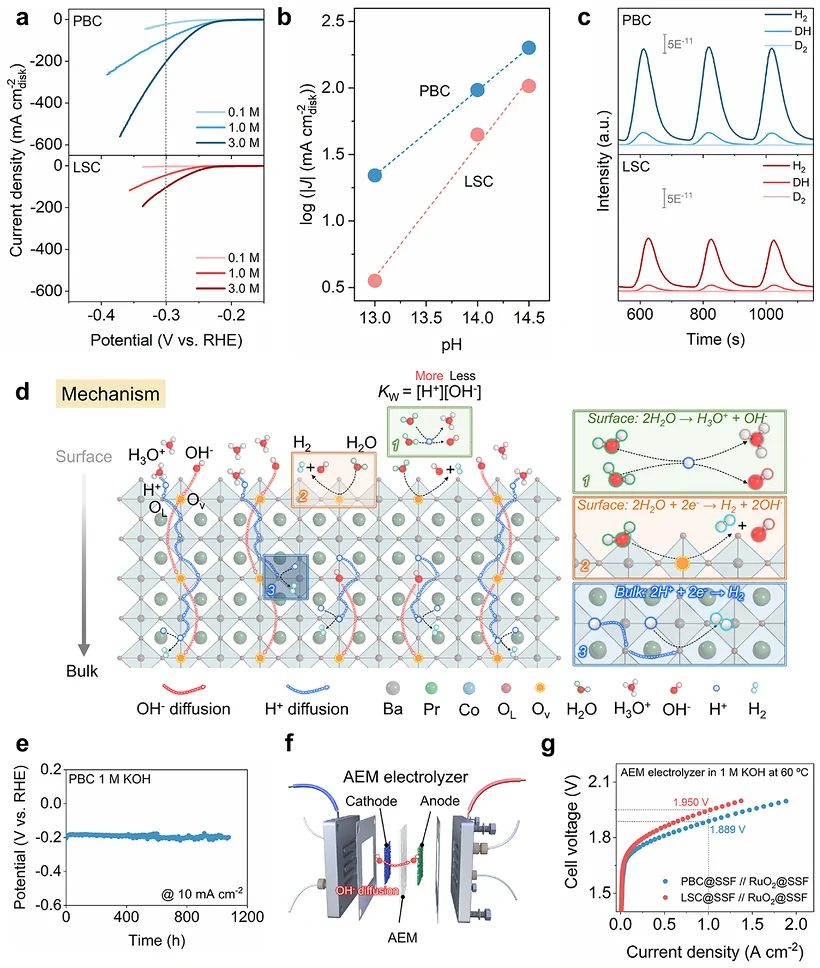
Bulk proton participation and catalysis mechanism. (a) The HER current densities of PBC and LSC under different alkaline solutions with different pH values. (b) The plot of log |*J*| as a function of pH values of different alkaline solutions. (c) The detected signals of H_2_ (*m/z* ≈ 2), DH (*m/z* ≈ 3), and D_2_ (*m/z* ≈ 4) from PBC and LSC during HER in D_2_O‐containing alkaline solutions. (d) The schematic diagram of the HER catalysis mechanism on PBC. (e) The HER stability of PBC at 10 mA cm^−2^ in 1 M KOH at room temperature. (f) The schematic diagram of OH^−^ diffusion in an AEM device. (g) The performance of AEM electrolyzers using PBC/LSC‐coated SSF as cathodes and commercial RuO_2_‐coated SSF as anodes in 1 M KOH at 60°C.

To explore whether H^+^ could diffuse into the bulk lattice of model oxides after HER, we conducted ToF‐SIMS with a probing depth of ∼120 nm. Obviously, PBC after HER shows a higher intensity of H^+^ signal (*m/z* ≈ 1) (Figure 3e) and a denser H^+^ spatial distribution than LSC after HER (Figure 3f). This indicates that H^+^ could also diffuse into the bulk lattice, and PBC exhibits a better bulk H^+^ diffusion capability because of the relatively stronger electrostatic interactions with H^+^ induced by its efficient bulk OH^−^ diffusion (bulk H^+^ and OH^−^ diffusion pathways in Figure 4d). To further support this finding, we track the signal and surface‐to‐bulk distribution of D ions (*m/z* ≈ 2) on model oxides after HER in D_2_O‐containing alkaline solutions. Similarly, the intensity of the D signal and the density of D spatial distribution in PBC after HER are higher than those in LSC after HER (Figure 3g,h), strengthening our conclusion.

Theoretically, we further calculated the adsorption energies of H^+^ diffusion in model oxides. DFT calculations show that the adsorption energies of H^+^ diffusion in PBC are lower than those in LSC (Figure 3i), suggesting a relatively facile bulk H^+^ diffusion in PBC. Furthermore, ELF maps display that the adsorption strength of H^+^ in PBC can be either strong (step 2) or weak (step 3) (Figure 3j), while that in LSC is consistently strong (Figure S13). We take the view that the weak H^+^ adsorption sites in PBC could benefit H^+^ diffusion, while the strong H^+^ adsorption sites may serve as active sites for H^+^ recombination into H_2_, which could trigger underlying bulk proton participation.

### 2.4 Bulk Proton Participation and Catalysis Mechanism

To probe the underlying bulk proton participation in model oxides, we performed electrochemical HER measurements in alkaline solutions with different pH values, operando isotope experiments, and operando Co‐*K* XANES spectroscopy. With increasing pH values, more OH^−^ species from the electrolyte could diffuse into the bulk lattice of the catalyst, inducing bulk H^+^ diffusion and participation via electrostatic interactions. The bulk H^+^ recombination into H_2_ could increase the current densities of H_2_ formation, resulting in pH‐dependent activity. Both PBC and LSC exhibit pH‐dependent HER activities (Figure 4a,b), implying the contribution of bulk H^+^ diffusion and participation. Benefiting from the superior bulk H^+^ diffusion capability, more diffused H^+^ can participate to form H_2_ in the PBC lattice, evidenced by its higher HER current densities in different solutions as compared with LSC (Figure 4a,b). Specifically, PBC delivers an overpotential of 303 mV at −100 mA cm^−2^ in 1 M KOH, which is 42 mV lower than that of LSC (345 mV). Moreover, PBC shows a smaller Tafel slope of 56.7 mV dec^−1^ than LSC (71.1 mV dec^−1^) in 1 M KOH (Figure S14a), indicating more favorable HER kinetics for PBC [46, 47]. As shown in Figure S14b, PBC achieves a mass activity of −415 mA mg^−1^ at −0.3 V versus RHE, approximately 2.2 times that of LSC (−192 mA mg^−1^).

To further verify the bulk H^+^ participation, we conducted operando isotope experiments on model oxides. It has been reported that an increased signal of DH/D_2_ gas product detected via mass spectroscopy on a catalyst in D_2_O‐containing solutions can demonstrate a stronger bulk proton participation on this catalyst during HER [18]. Of note, the detected signals of all gas products (i.e., H_2_, DH, and D_2_) from PBC in D_2_O‐containing alkaline solutions during consecutive HER cycles are significantly higher than those from LSC (Figure 4c), corroborating the more active involvement of bulk protons in PBC. As bulk H^+^ participation proceeds, the bulk Co ions in a catalyst will undergo reduction during HER, serving as another evidence to prove bulk H^+^ participation. Relative to LSC, the reduction of bulk Co ions in PBC is stronger, as evidenced by the bulk‐sensitive operando Co‐*K* XANES spectra during HER (Figure S15), reinforcing our conclusions. The favorable bulk H^+^ participation extends the HER regions from the surface to the bulk of the catalyst to boost HER (point 3 in Figure 4d).

In addition to the surface‐to‐bulk diffusion, induction, and reaction processes of OH^−^ and H^+^ species, the behaviors of H_2_O reactants in alkaline HER should also be clarified. From DFT computations, we found that relatively large H_2_O reactants cannot diffuse into the lattice of model oxides like OH^−^ and H^+^ because of the structural steric hindrance. Moreover, no signals of H_2_O^18^ or D_2_O (*m/z* ≈ 20) are observed in ToF‐SIMS plots for PBC after HER in H_2_O^18^‐ or D_2_O‐containing alkaline solutions (Figure S16). This means that the H_2_O dissociation step is confined to the surface of the catalyst (point 2 in Figure 4d). Furthermore, we calculated the surface H_2_O dissociation step (Volmer step: H_2_O → H^*^ + OH^*^) on model oxides and found that PBC with lower energy barriers (Figure S17) enables more facile and favorable dissociation of surface H_2_O to provide more H^+^ sources for subsequent bulk H^+^ diffusion and participation.

We now can clearly elucidate the surface‐to‐bulk behaviors of OH^−^, H^+^, and H_2_O species and related mechanisms on the oxygen‐vacancy‐ordered PBC for efficient alkaline HER (Figure 4d). First, abundant OH^−^ ions from alkaline electrolyte diffuse from the surface to the bulk of PBC through the short pathways created by its ordered oxygen vacancies (*O*_V_). Then, surface OH^−^ concentration on PBC is reduced because of its efficient bulk OH^−^ diffusion into the lattice. The decreased surface OH^−^ concentration drives the surface water ionization equilibrium toward products (2H_2_O ↔ H_3_O^+^ + OH^−^) under Le Chatelier's principle, thus enriching surface H_3_O^+^ species to generate a unique acid‐like surface environment for enhancing surface HER kinetics (point 1 in Figure 4d). Next, the protons from surface H_3_O^+^ species and surface H_2_O dissociation (point 2 in Figure 4d) diffuse into the bulk lattice of PBC via the channels of lattice oxygen sites (*O*_L_) under electrostatic interactions with bulk OH^−^ diffusion. The bulk *O*_L_ sites with strong H^+^ adsorption recombine the diffused H^+^ into H_2_ (point 3 in Figure 4d), triggering bulk H^+^ participation to improve HER activity.

We further measured the HER stability of PBC in 1 M KOH, which delivers stable operation of over 1000 h at 10 mA cm^−2^ (Figure 4e). In the AEM device, since the OH^−^ diffuses from the cathode across the membrane and further to the anode reaction (Figure 4f), the strong bulk OH^−^ diffusion capability of the cathode should theoretically improve the AEM electrolyzer performance and enhance the device stability. As anticipated, the AEM cell with PBC‐coated Ni foam as the cathode and RuO_2_‐coated Ni foam as the anode in 1 M KOH at 80°C substantially outperforms the combination of LSC‐coated Ni foam as the cathode and RuO_2_‐coated Ni foam as the anode (Figure S18a). The constructed AEM cell using PBC with efficient OH^−^ diffusion capability as the cathode also exhibits good cell stability at 100 mA cm^−2^ (Figure S18b).

To further evaluate the AEM electrolyzers’ performance and stability under high current densities (i.e., 1 A cm^−2^), we sprayed the catalysts on stainless steel felts (SSF) and Ni felts, which possess better mechanical strength than Ni foams. As shown in Figure 4g, the oxygen‐vacancy‐ordered PBC‐coated SSF cathode with superior OH^−^ conductivity still delivers better AEM electrolyzer performance (1.889 V at 1 A cm^−2^) than the oxygen‐vacancy‐disordered LSC‐coated SSF cathode (1.950 V at 1 A cm^−2^). Similarly, the AEM electrolyzer using a PBC‐coated Ni felt cathode (1.870 V at 1 A cm^−2^) is superior to the cell with an LSC‐coated Ni felt cathode (1.931 V at 1 A cm^−2^), as presented in Figure S19. Although the performance of AEM electrolyzers employing PBC cathodes is lower than that of noble‐metal‐based Pt/C cathodes (Figure S19), the PBC‐based cathode still exhibits superior performance compared with many recently reported representative AEM cathodes (Figure S19 and Table S3). Noteworthily, the AEM electrolyzer constructed with a PBC‐coated SSF cathode can operate stably at 1 A cm^−2^ for over 100 h with a low degradation rate of only 0.26 mV h^−1^, surpassing the cell using an LSC‐coated SSF cathode with a large degradation rate of 15.72 mV h^−1^ within only 4 h (Figure S20). The ordered oxygen vacancies in PBC provide efficient pathways for OH^−^ diffusion, enabling the PBC cathode to promote OH^−^ transport from the cathode side toward the anode through the membrane. As a result, the PBC‐based AEM electrolyzer achieves enhanced performance and stable operation at 1 A cm^−2^.

To further examine whether the proposed mechanism remains operative after stability testing, ToF‐SIMS, operando Raman, and operando isotope labeling measurements were conducted on PBC/LSC‐coated SSF cathodes after AEM electrolyzer stability testing at 1 A cm^−2^. To probe the diffusion behaviors of OH^−^ and protons, the PBC/LSC‐coated SSF cathodes after stability testing were activated in D_2_O‐containing alkaline solutions, and the intensities of OH^−^ and D signals were subsequently compared by ToF‐SISM. As illustrated in Figure S21, the PBC‐coated SSF cathode exhibits stronger OH^−^ and D signal intensities than the LSC‐coated SSF cathode after stability testing, demonstrating the retained superior OH^−^ and proton diffusion capabilities of the PBC cathode after stability operation. To examine whether bulk OH^−^ diffusion induces surface H_3_O^+^ enrichment under Le Chatelier's principle, operando Raman measurements were performed on PBC/LSC‐coated SSF cathodes after stability testing. Operando Raman results further reveal that, as the HER overpotential increases, the PBC‐coated SSF cathode still shows a distinct H_3_O^+^ characteristic band at ∼1700 cm^−1^ (Figure S22a), indicating its retained acid‐like surface local environment even after long‐term operation. By contrast, no such band is observed for the LSC‐coated SSF cathode after stability testing (Figure S22b). To further probe the involvement of bulk protons, we performed operando isotope experiments on PBC/LSC‐coated SSF cathodes after stability testing. In D_2_O‐containing alkaline electrolytes, the PBC‐coated SSF cathode exhibits markedly stronger signals for all detected HER‐related gaseous products (namely, H_2_, DH, and D_2_) than the LSC‐coated SSF cathode (Figure S23), confirming the more active participation of bulk protons in PBC even after stability testing. Therefore, the mechanism proposed above remains valid after stability testing (Figure 4d).

Considering the possible effects of OH^−^‐conduction pathways formed by the ionomer itself and by the interfaces between the ionomer and catalyst on AEM electrolyzer performance, we replaced the OH^−^‐conducting ionomer on the cathode side with polytetrafluoroethylene (PTFE), which has no ionic conductivity and only provides physical adhesion. As shown in Figure S24, replacing OH^−^‐conducting ionomer with PTFE as the cathode‐side binder leads to decreased AEM electrolyzer performance, which can be attributed to the absence of OH^−^‐conduction pathways originating from the ionomer itself and the possible ionomer‐catalyst interfaces. However, the performance decrease is relatively limited, implying that the intrinsic OH^−^ conductivity of the cathodes themselves still plays an indispensable role in AEM electrolyzer performance. The possible contribution of OH^−^‐conduction pathways at the ionomer‐catalyst interfaces requires further investigation in future studies.

## 3 Conclusion

By combining multiple operando/ex situ characterizations and computations, for the first time, we shed light on the diffusion, induction, and reaction behaviors of OH^−^, H^+^, and H_2_O species on oxygen‐vacancy‐ordered and oxygen‐vacancy‐disordered model oxides in alkaline HER. Importantly, we first demonstrate that bulk OH^−^ diffusion (the abundant ions in electrolyte) significantly affects the generation of proton sources and the diffusion/reaction behaviors of protons, thus governing the performance of alkaline HER and AEM devices. The relationships between oxygen‐vacancy properties (ordering and content) and bulk OH^−^ diffusion capabilities of model oxides have been successfully established. Given that bulk OH^−^ diffusion may also occur and play a critical role in many solution‐phase reactions (such as the oxygen evolution reaction, CO_2_ electroreduction, and small organic molecule oxidation), our work is expected to provide valuable guidance for a broad range of electrochemical reactions and applications.

## Author Contributions

**Xiao Sun**: data curation, formal analysis, and methodology. **Daqin Guan**: conceptualization, investigation, funding acquisition, writing – original draft, methodology, validation, visualization, writing – review and editing, software, formal analysis, project administration, data curation, supervision, and resources. **Hengyue Xu**: software, formal analysis, data curation, and resources. **Chun‐Kuo Peng**: methodology, formal analysis, and data curation. **Leqi Zhao**: data curation, formal analysis, and methodology. **Yu‐Cheng Huang**: methodology, formal analysis, and data curation. **Zongping Shao**: supervision, funding acquisition, investigation, conceptualization, and project administration. **Nai Shi**: methodology, formal analysis, and data curation. **Zezhou Lin**: visualization. **Xiaomin Xu**: formal analysis and data curation. **Chao Jing**: methodology, formal analysis, and data curation. **Jiayi Tang**: data curation, formal analysis, and methodology. **Zhiwei Hu**: data curation, formal analysis, methodology, and resources.

## Funding

Australian Research Council (ARC) Discovery Early Career Researcher Award (no. DE250101434, D.G.); ARC Discovery Project (no. DP250104303, Z.S.).

## Conflicts of Interest

The authors declare no conflicts of interest.

## Supporting information




**Supporting File**: adma74662‐sup‐0001‐SuppMat.docx.

## Data Availability

The data that support the findings of this study are available from the corresponding author upon reasonable request.
