# Supplementary material for: Efficient Hydroxyl Diffusion Triggers Surface Hydronium Enrichment and Bulk Proton Participation to Boost Alkaline Hydrogen‐Evolving Reaction
Source: Adv Mater. 2026 Aug 17;38(57):e74662. doi: 10.1002/adma.74662 (PMC13660816; doi:10.1002/adma.74662)
Supplement: Supplementary file 1 [file ADMA-38-e74662-s001.docx]

Supporting Information

Efficient Hydroxyl Diffusion Triggers Surface Hydronium Enrichment and Bulk Proton Participation to Boost Alkaline Hydrogen-Evolving Reaction

Daqin Guan,* Hengyue Xu, Xiao Sun, Leqi Zhao, Chun-Kuo Peng, Chao Jing, Yu-Cheng Huang, Jiayi Tang, Nai Shi, Zezhou Lin, Xiaomin Xu, Zhiwei Hu, and Zongping Shao*

**1. Experimental Section**

**Materials**

Both PBC and LSC perovskite oxides were prepared via the conventional sol-gel approach. In brief, the stoichiometric related nitrates (Pr(NO_3_)_3_•6H_2_O, Ba(NO_3_)_2_, Co(NO_3_)_2_•6H_2_O, La(NO_3_)_3_•6H_2_O, or Sr(NO_3_)_2_) bought from Sigma-Aldrich company were weighed and dissolved in pure water. Then, the ethylenediaminetetraacetic acid (EDTA) and citric acid (CA) purchased from ChemSupply company were weighed and dissolved as the complexing agents in a molar ratio of 1:2:1 for EDTA/CA/total metal ions of the oxide. The 28 wt% ammonia solution bought from ChemSupply company was used to control the pH of solution to 6~7. The mixed solutions were heated at ~100 °C with continuous stirring to produce a gel. A solid precursor was obtained after calcining the gel at 250 °C in air for 10 h. The solid precursor was further calcined at 1000 °C in air for 10 h to yield the final powders of PBC and LSC. The RuO_2_ catalyst and D_2_O (99.9 atom% D) used in this work were purchased from Sigma-Aldrich company. The H_2_O^18^ with ≥98 atom% O^18^ was bought from Taiyo Nippon Sanso company.

***Ex-situ* characterizations**

Laboratory XRD was measured on a Bruker D8A diffractometer with a Cu X-ray source of λ = 1.5418 Å, followed by XRD refinement using the TOPAS software. High-resolution SXRPD was conducted at the TLS 01C2 beamline with λ = 0.6199 Å in NSRRC of Taiwan. The spherical-aberration-corrected HAADF, ABF, and atomic mapping images were collected on a JEOL ARM 200F microscope under an acceleration voltage of 200 kV. The soft XAS (Co-*L*_3_ and O-*K* edges in TEY mode) and hard XAS (Co-*K* XANES and EXAFS in transmission mode) spectra were acquired at the TLS 11A and TPS 44A beamlines, respectively. The CoO, NiO, and Co single crystals were simultaneously measured to calibrate the photon energies for Co-*L*_3_, O-*K*, and Co-*K* XAS spectra, respectively. Co-*K* XANES and EXAFS spectra were analyzed by the ATHENA software packages [1]. EPR was performed on a Bruker EMXplus-6/1 spectrometer. XPS was taken on a Kratos AXIS Ultra DLD system, where the fitting was proceeded using the CASA XPS software. ToF-SIMS results were achieved on an IONTOF M6 equipment. To obtain good signals of sputtering O 1*s* XPS after HER and ToF-SIMS after HER, samples were prepared on the flat and conductive glassy carbon films with a loading of ~0.25 mg cm^-2^ and operated at -0.2 V vs. RHE in 1.0 M KOH for 5 h.

**OH^-^ conductivity measurements**

The sample powder (0.4 g) was thoroughly ground with three drops of 5 wt% PVA, and pressed into pellets with 15 mm diameter. The pressed pellets were then calcined in air at 1050 °C for 5 h to form dense pellets. Ag wires and Ag paste were used as the current collectors, where the effective area is 0.5 cm^2^. The ohmic resistance was measured in a home-made setup via a Zahner Zennium electrochemical workstation in 1.0 M KOH at 25 °C.

***Operando* characterizations**

*Operando* FTIR was conducted on a PerkinElmer FTIR spectrometer in 1.0 M KOH at 25 °C, where the samples were prepared on a Si crystal coated with a gold layer as the working electrode. *Operando* Raman was performed on a Horiba LabRAM HR Evolution confocal microscope in 1.0 M KOH at 25 °C, where the samples were prepared on the glassy carbon. A sealed H-type electrolyzer was used for the *operando* isotope experiments in D_2_O-containing 1.0 M KOH alkaline solutions at 25 °C, where Ar gas was applied as the carrier gas to mass spectroscopy for product analysis. The samples were prepared on the glassy carbon as the working electrodes and operated under consecutive HER cycles, where mass spectroscopy detected the online product signals. For *operando* XANES in 1.0 M KOH at 25 °C, carbon papers were used as the working electrodes for sample preparation.

**Electrochemical HER and AEM electrolyzer measurements**

The electrochemical HER tests were performed in a three-electrode rotating disk (2400 rpm) electrode configuration (Pine Research Instrumentation) using CHI 760E potentiostat in Ar-saturated KOH solutions at 25 °C, where 0.196 cm^2^ glassy carbon, Hg/HgO, and graphite rod served as the working, reference, and counter electrodes, respectively. 5 μL homogenous catalyst ink from the mixture of 10 mg oxide, 2 mg carbon black, 0.1 mL 5 wt% Nafion solution, and 1.0 mL absolute ethanol was dropped on the glassy carbon for a working electrode with a catalyst loading of 0.232 mg cm^-2^. For the catalyst-coated Ni foam electrodes, AEM electrolyzer tests were assessed in 2 × 2 cm^2^ electrolyzers in 1.0 M KOH at 80 °C. For the cathode ink, the model oxides were mixed with carbon black and anion exchange ionomer (Fumion FAA-3-SOLUT-10) at a mass ratio of 5:1:5 in a mixture of isopropanol/H_2_O solvent. For the anode ink containing commercial RuO_2_ (Sigma), no carbon black was added. The homogenous catalyst ink after ultrasonication was sprayed on the Ni foam with a catalyst loading of 1 mg cm^-2^ for both cathodes and anodes. The cathode inks containing PBC or LSC and the anode ink containing commercial RuO_2_ for the catalyst-coated SSF/Ni felt electrodes (2 × 2 cm^2^) were prepared using a procedure similar to that used for Ni foam electrodes. No carbon black was added for the cathode ink containing commercial Pt/C (60 wt%, Fuel Cell Store). The catalyst loading on SSF and Ni felts was 2 mg cm^-2^ for both the cathodes and anodes. The NiMoO_x_@Ni felt cathode was prepared following a previously reported hydrothermal reaction method [2]. The performance and stability of the SSF/Ni felt-based AEM electrolyzers were evaluated in 1.0 M KOH at 60 °C. PiperIon-A40 bought from Fuel Cell Store was used as the anion exchange membrane.

**DFT calculations**

All spin-polarized DFT computations were performed via the Vienna Ab initio Simulation Package [3] with the projector-augmented wave approach [4]. The generalized gradient approximation of Perdew–Burke–Ernzerhof [5] was adopted for the exchange-correlation interactions. A Hubbard correction with an effective *U* value of 3.3 eV was applied for Co-based model oxides here under the DFT + *U* method [6]. The Grimme DFT–D3 scheme was used for the long-range dispersion forces [7]. The plane-wave kinetic energy cutoff was set to 500 eV, ensuring the total-energy convergence to within 2 × 10^-5^ eV. Brillouin-zone integrations were performed using a *Γ*-centered Monkhorst–Pack mesh of 2 × 2 × 1 for slab models. A ~15 Å vacuum spacing was also applied. Half of the bottom layers were fixed to the bulk environment, while the remaining atoms were fully relaxed until the residual forces were below 0.05 eV Å^-1^. All optimized structures correspond to local minima with converged forces and total energies.

**2. Supplementary figures**

**Figure S1.** XRD refinements for PBC and LSC.

**Figure S2.** HAADF image and atomic mapping for PBC along [10$\bar{1}$] zone axis.

**Figure S3.** The spherical-aberration-corrected ABF image, HAADF image, and atomic mapping for LSC.

**Figure S4.** Co 2*p* XPS spectra of PBC and LSC.

**Figure S5.** O 1*s* XPS spectra of PBC and LSC.

**Figure S6.** Co-*K* XANES spectra of PBC and LSC, where EuCoO_3_ and BaCoO_3_ serve as the pure Co^3+^ and pure Co^4+^ standards [6, 8, 9], respectively.

**Figure S7.** (a) The prepared dense oxide pellet with OH^-^-conducting AEM on both sides for OH^-^ conductivity measurement. The measured ohmic resistance of (b) LSC and (c) PBC.

**Figure S8.** The fitting results of sputtering O 1*s* XPS spectra of PBC after HER.

**Figure S9.** The fitting results of sputtering O 1*s* XPS spectra of LSC after HER.

**Figure S10.** ELF plots of the adsorption of OH^-^ from the surface to the bulk for LSC.

**Figure S11.** *Operando* FTIR spectra of PBC during HER.

**Figure S12.** *Operando* FTIR spectra of LSC during HER.

**Figure S13.** ELF plots of the adsorption of H^+^ from the surface to the bulk for LSC.

**Figure S14.** (a) Tafel values and mass activities for PBC and LSC in 1 M KOH.

**Figure S15.** (a) *Operando* Co-*K* XANES spectra of PBC and LSC during HER. (b) Enlarged regions of the normalized absorption of ~0.8 in the *operando* Co-*K* XANES spectra for PBC and LSC.

**Figure S16.** ToF-SIMS results of PBC after HER in (a) H_2_O^18^-containing and (b) D_2_O-containing alkaline solutions. (c) The schematic diagram of the structural steric hindrance for H_2_O species.

**Figure S17.** The calculated surface H_2_O dissociation energies of (a) PBC and (b) LSC.

**Figure S18.** (a) The performance of AEM electrolyzers using PBC/LSC-coated Ni foams as cathodes and commercial RuO_2_-coated Ni foams as anodes in 1 M KOH at 80 °C. (b) The stability of AEM electrolyzer using PBC-coated Ni foam as cathode and commercial RuO_2_-coated Ni foam as anode at 100 mA cm^-2^ in 1 M KOH at 80 °C.

**Figure S19.** The performance of the AEM electrolyzers using PBC/LSC/Pt/C/NiMoO_x_-coated Ni felts as cathodes and commercial RuO_2_-coated Ni felts as anodes in 1 M KOH at 60 °C.

**Figure S20.** The stabilities of the AEM electrolyzers using PBC/LSC-coated SSF as cathodes and commercial RuO_2_-coated SSF as anodes under 1 A cm^-2^ in 1 M KOH at 60 °C.

**Figure S21.** ToF-SIMS peak signals of (a) OH^-^ and (b) D species for PBC/LSC-coated SSF cathodes after AEM electrolyzer stability testing.

**Figure S22.** *Operando* Raman results for (a) PBC- and (b) LSC-coated SSF cathodes after AEM electrolyzer stability testing.

**Figure S23.** *Operando* isotope experiments on (a) PBC- and (b) LSC-coated SSF cathodes after AEM electrolyzer stability testing.

**Figure S24.** The performance of the AEM electrolyzers using PBC/LSC-coated Ni felts as cathodes and commercial RuO_2_-coated Ni felts as anodes in 1 M KOH at 60 °C. For the PBC/LSC@Ni felt + PTFE // RuO₂@Ni felt cell configurations, PTFE was employed as the binder on the cathode side.

**3. Supplementary tables**

**Table S1.** Results of XRD refinements.

| Sample | Space group | *a* (Å) | *b* (Å) | *c* (Å) | GOF^a^ |
| --- | --- | --- | --- | --- | --- |
| PBC | *P*4/*mmm* | 7.803 | 7.803 | 7.638 | 1.06 |
| LSC | *Pm*-3*m* | 3.839 | 3.839 | 3.839 | 1.04 |

^a^GOF stands for the goodness of fitting. An acceptable GOF value for a good XRD refinement result should be lower than 2 as reported [6, 9].

**Table S2.** The fitting results of *k*^3^-weighted EXAFS spectra.

| Samples | Bond type | CN^a^ | R  (Å)^b^ | σ^2^  (10^-3^×Å^2^)^c^ | R* factor^d^ |
| --- | --- | --- | --- | --- | --- |
| PBC | Co–O | 5.75 | 1.96 | 10.13 | 0.0019 |
| LSC | Co–O | 5.89 | 1.93 | 7.89 | 0.017 |

^a^CN represents the coordination number.

^b^R stands for the interatomic distance with phase correction.

^c^σ^2^ means Debye-Waller factor.

^d^R* factor is a standard to weight the quality of fitting.

**Table S3.** Comparisons of the performance of representative AEM electrolyzers in 1 M KOH.

| Cathode//Anode | Temperature  (°C) | Voltage (V)  at 1 A cm^-2^ | References |
| --- | --- | --- | --- |
| PBC perovskite@Ni felt//RuO_2_@Ni felt | 60 | 1.87 | This work |
| LSC perovskite@Ni felt//RuO_2_@Ni felt | 60 | 1.93 | This work |
| PBC perovskite@SSF//RuO_2_@SSF | 60 | 1.89 | This work |
| LSC perovskite@SSF//RuO_2_@SSF | 60 | 1.95 | This work |
| CdNNi_3_ antiperovskite@Ni felt//NiFeO_x_@Ni felt | 60 | 1.90 | [10] |
| In_0.9_NNi_3_ antiperovskite@Ni felt//NiFeO_x_@Ni felt | 60 | 1.92 | [11] |
| CdNNi_3_ antiperovskite@Ni foam//Fe-CdNNi_3_@Ni foam | 80 | 1.96 | [12] |
| Pt/C@Ni foam//RP/SP perovskite composite@Ni foam | 60 | ~1.97 | [13] |
| Pt/C@Ni substrate//BZCC perovskite@ Ni substrate | 40 | 2.07 | [14] |
| Pt_T_/CoOOH//NiFe LDH | 80 | 1.90 | [15] |
| Pt/C//Ni-TDC_0.6_BDC_0.4_ | 60 | 1.95 | [16] |
| Ni_4_Mo@PDA//NiFe LDH | 70 | ~1.92 | [17] |
| PtRuFeCoNiCu HEA//FeCoNiCrRu HEO | 60 | 2.03 | [18] |
| Pt/C//CoCrO_x_ | 60 | ~1.98 | [19] |
| Ir@Zr-CoP//Ir@Zr-CoP | 60 | 1.88 | [20] |
| Ru_SA_/d-Mo_2_C//IrO_2_ | 60 | 1.89 | [21] |

**References**

[1]	B. Ravel, and M. Newville, "Athena, Artemis, Hephaestus: Data Analysis for X-Ray Absorption Spectroscopy Using IFEFFIT," *Journal of Synchrotron Radiation* 12 (2005): 537, <https://doi.org/10.1107/S0909049505012719>.

[2]	J. Zhang, T. Wang, P. Liu et al., "Efficient Hydrogen Production on MoNi_4_ Electrocatalysts with Fast Water Dissociation Kinetics," *Nature Communications* 8 (2017): 15437, <https://doi.org/10.1038/ncomms15437>.

[3]	G. Kresse, and J. Furthmüller, "Efficient Iterative Schemes for *Ab Initio* Total-Energy Calculations Using a Plane-Wave Basis Set," *Physical Review B* 54 (1996): 11169, <https://doi.org/10.1103/PhysRevB.54.11169>.

[4]	P. E. Blӧchl, "Projector Augmented-Wave Method," *Physical Review B* 50 (1994): 17953, <https://doi.org/10.1103/PhysRevB.50.17953>.

[5]	M. Ernzerhof, and G. E. Scuseria, "Assessment of the Perdew–Burke–Ernzerhof Exchange-Correlation Functional," *The Journal of Chemical Physics* 110 (1999): 5029, <https://doi.org/10.1063/1.478401>.

[6]	D. Guan, H. Xu, Q. Zhang et al., "Identifying a Universal Activity Descriptor and a Unifying Mechanism Concept on Perovskite Oxides for Green Hydrogen Production," *Advanced Materials* 35 (2023): 2305074, <https://doi.org/10.1002/adma.202305074>.

[7]	S. Grimme, J. Antony, S. Ehrlich, and H. Krieg, "A Consistent and Accurate *Ab* *Initio* Parametrization of Density Functional Dispersion Correction (DFT-D) for the 94 Elements H-Pu," *The Journal of Chemical Physics* 132 (2010): 154104, <https://doi.org/10.1063/1.3382344>

[8]	S. Agrestini, K. Chen, C. Y. Kuo et al., "Nature of the Magnetism of Iridium in the Double Perovskite Sr_2_CoIrO_6_," *Physical Review B* 100 (2019): 014443, <https://doi.org/10.1103/PhysRevB.100.014443>.

[9]	D. Guan, K. Zhang, Z. Hu et al., "Exceptionally Robust Face-Sharing Motifs Enable Efficient and Durable Water Oxidation," *Advanced Materials* 33 (2021): 2103392, <https://doi.org/10.1002/adma.202103392>.

[10]	Y. Chen, Z. Tang, Z. Liu et al., "Toward the Ideal Alkaline Hydrogen Evolution Electrocatalyst: A Noble Metal-Free Antiperovskite Optimized with A-site Tuning," *Advanced Materials* 37 (2025): 2504607, <https://doi.org/10.1002/adma.202504607>.

[11]	Y. Chen, Z. Liu, B. Chen et al., "A‐site Cation Deficiency in Antiperovskites for Precisely Accelerating the Volmer Step of Alkaline Hydrogen Evolution," *Advanced Energy Materials* 15 (2025): e03319, <https://doi.org/10.1002/aenm.202503319>.

[12]	J. Zhang, Y. Tu, L. Zhang et al., "Surface-Reconstructed CdNNi_3_ Antiperovskite Electrocatalyst: Unlocking Ampere-Level Current Density for Hydrogen Evolution," *ACS Nano* 18 (2024): 32077, <https://doi.org/10.1021/acsnano.4c10486>.

[13]	J. Tang, X. Xu, T. Tang, Y. Zhong, and Z. Shao, "Perovskite-Based Electrocatalysts for Cost-Effective Ultrahigh-Current-Density Water Splitting in Anion Exchange Membrane Electrolyzer Cell," *Small Methods* 6 (2022): 2201099, <https://doi.org/10.1002/smtd.202201099>.

[14]	L. Natarajan, T. T. T. Nga, M. Kwon et al., "Redox-Engineered Co-Cu-Zr Perovskites for Durable Anion Exchange Membrane Electrolysis," *Small* 21 (2025): e09516, <https://doi.org/10.1002/smll.202509516>.

[15]	P. Ma, J. Xu, Q. Hao et al., "Site-Dependent Hydrogen Adsorption of Pt Single Atoms for Ampere-Level Alkaline Hydrogen Evolution," *Angewandte Chemie International Edition* (2026): e5990615, <https://doi.org/10.1002/anie.5990615>.

[16]	Z. Huang, B. Wu, S. Zhang et al., "Spin-State Engineering of Ni Centers by Dual-Ligand Competitive Coordination for Superior Oxygen Evolution Reaction," *Angewandte Chemie International Edition* 65 (2026): e9878440, <https://doi.org/10.1002/anie.9878440>.

[17]	F. Kong, J. Li, Z. Hou et al., "Polydopamine Nanocages Orchestrate Multi-Site Networks on the Ni_4_Mo Electrocatalyst for Efficient and Ultrastable Hydrogen Evolution," *Journal of the American Chemical Society* 148 (2026): 15771, <https://doi.org/10.1021/jacs.5c21810>.

[18]	Z. X. Hui, Z. Ju, J. Z. Li et al., "Dual-Mechanism Synergy of Adsorbate Evolution and Lattice Oxygen in High-Entropy Oxides for Enhanced Water Oxidation," *Energy & Environmental Science* (2026), <https://doi.org/10.1039/d6ee00598e>.

[19]	S. Li, T. Liu, W. Zhang et al., "Highly Efficient Anion Exchange Membrane Water Electrolyzers Via Chromium-Doped Amorphous Electrocatalysts," *Nature Communications* 15 (2024): 3416, <https://doi.org/10.1038/s41467-024-47736-0>.

[20]	Q. P. Ngo, T. T. Nguyen, Q. T. T. Le, J. H. Lee, and N. H. Kim, "Unveiling the Synergistic Effect of Atomic Iridium Modulated Zirconium‐Doped Pure Phase Cobalt Phosphide for Robust Anion‐Exchange Membrane Water Electrolyzer," *Advanced Energy Materials* 13 (2023): 2301841, <https://doi.org/10.1002/aenm.202301841>.

[21]	W. Zhou, J. Wu, Y. Cai et al., "Optimizing the Interfacial Water Network Via Electron‐Enriched Ru Single Atoms for Ultrafast Hydrogen Evolution Kinetics," *Advanced Functional Materials* (2026): e75469, <https://doi.org/10.1002/adfm.75469>.
